# Progressive myoclonic ataxia as an initial symptom of typical type I sialidosis with *NEU1* mutation

**DOI:** 10.1002/acn3.52212

**Published:** 2024-10-31

**Authors:** Jingjing Lin, Yun‐Lu Li, Bo‐Li Chen, Hui‐Zhen Su, Yi‐Heng Zeng, Rui‐Huang Zeng, Yu‐Duo Zhang, Ru‐Kai Chen, Nai‐Qing Cai, Yi‐Kun Chen, Ru‐Ying Yuan, Jun‐Yi Jiang, Xiang‐Ping Yao, Ning Wang, Wan‐Jin Chen, Kang Yang

**Affiliations:** ^1^ Department of Neurology and Institute of Neurology of First Affiliated Hospital Institute of Neuroscience, and Fujian Key Laboratory of Molecular Neurology, Fujian Medical University Fuzhou 350005 China

## Abstract

**Objective:**

Expand genetic screening for atypical Type I sialidosis (ST‐1) could address its underdiagnosed in both progressive myoclonic ataxia (PMA) and ataxia patients. To evaluate the potential founder effect of mutation in the population.

**Methods:**

We enrolled 231 patients with PMA or ataxia from the First Affiliated Hospital of Fujian Medical University. Through Whole Exome Sequencing and Sanger sequencing, we identified the causative gene in patients. Haplotype analysis was employed to explore a potential founder effect of the *NEU1* c.544A>G mutation.

**Results:**

A total of 31 patients from 23 unrelated families were genetically diagnosed with ST‐1. A significant 80.6% of these patients were homozygous for the c.544A>G mutation. We discovered six different *NEU1* variants, including two novel mutations: c.951_968del and c.517T>G. The mean age of onset was 18.0 ± 7.1 years. The clinical spectrum of ST‐1 featured ataxia and myoclonus as the most common initial symptoms. Over 40% suffered from controlled generalized tonic–clonic seizures. Mobility and independence varied greatly across the cohort. Cherry‐red spots were rare, occurring in just 9.5% (2/21) of patients. Brain MRIs were typically unremarkable, except for two patients with unusual findings. EEGs showed diffuse paroxysmal activity in 17 patients. The c.544A>G mutation in *NEU1* is a founder variant in Fujian, with a unique haplotype prevalent in East Asians.

**Interpretation:**

ST‐1 should be suspected in patients with PMA or ataxia in Southeast China, even without macular cherry‐red spots and seizures, and the premier test could be a variant screening of the founder variant *NEU1* c.544A>G.

## Introduction

Sialidosis is a rare autosomal recessive lysosomal storage disease.^1^ According to the onset age and severity, sialidosis can be classified into two clinical types. Compared with type II sialidosis, type I sialidosis (ST‐1) is a late‐onset milder form, characterized by macular cherry‐red spots, visual impairment, myoclonus, ataxia, and seizures.^2^ With fewer than a hundred patients reported in the world, the clinical data of ST‐1 is still limited.^3^ ST‐1 has been known as “cherry‐red spot myoclonus syndrome” for decades,^4^ but recently some studies showed that typical features could be absent in patients.^1^, ^5^, ^6^, ^7^, ^8^, ^9^, ^10^ Some patients may present with progressive myoclonic ataxia (PMA) without the classic cherry‐red spot on the fundus. The initial symptoms can be highly variable, including ataxia, myoclonus, or a decline in vision. This variability indicates that the clinical manifestations of ST‐1 are complex, making diagnosis based solely on clinical presentation challenging and highly susceptible to being overlooked in clinical practice. Therefore, it is crucial to have a thorough understanding of the disease in a clinical setting before proceeding with genetic testing to establish a definitive diagnosis.

The pathogenic gene for ST‐1 is *NEU1*, which includes six exons and encodes the enzyme sialidase. *NEU1*, also known as sialidase, plays a critical enzymatic role in the cleavage of sialic acid residues from sialoglycoconjugates.^11^, ^12^ Mutations in the *NEU1* gene can lead to a deficiency of neuraminidase, disrupting the normal breakdown of sialic acid‐containing molecules. As a result, large sialic acid‐rich molecules accumulate within cells, while sialic acid‐laden oligosaccharides are excreted via the kidneys. This aberrant sialic acid buildup within the body can lead to a wide range of clinical symptoms. Pathogenic mutations have been identified across all exons of *NEU1*, with those in exons 4 and 5 being notably prevalent. A particularly common mutation in East Asian populations is the c.544A>G (p.S182G) variant in the *NEU1* gene, which is less frequent among Europeans.^3^ The status of c.544A>G as a potential founder mutation within the Chinese population remains to be clarified.

Here, we describe the diagnostic process for ST‐1 in 31 atypical patients from a cohort with PMA and ataxia, further summarizing their clinical features and confirming a founder effect in Southeast China. Our findings contribute to a broader understanding of the genetics and clinical aspects of ST‐1, which may be valuable for guiding clinical decisions and enhancing the accuracy of clinical diagnoses.

## Patients and Methods

### Patient enrollment

Initially, a total of 231 patients diagnosed with PMA or progressive ataxia were recruited in this study from the Department of Neurology of the First Affiliated Hospital of Fujian Medical University over the last 13 years (January 2010–December 2023). The criteria for the diagnosis of PMA were as follows: (a) the presence of myoclonus, (b) the presence of ataxia, and (c) no or infrequent epilepsy (treatment‐responsive epilepsy including all types of epileptic seizures).^13^ Patients presenting with progressive ataxia (spinocerebellar ataxia‐linked repeat expansions had previously been excluded) were included. We also recruited 200 individuals without neurological symptoms as healthy controls. This project was approved by the Ethics Committee for Medical Research of the First Affiliated Hospital of Fujian Medical University. Informed consent from all participants was obtained according to the Declaration of Helsinki.

### Clinical information

Information on clinical characteristics, laboratory results, echocardiography, and electrophysiological examinations was collected. Patients with confirmed diagnosis had received detailed neurological examinations performed by at least two senior neurologists. Some auxiliary examinations were performed for clinical purposes: Brain MRI, electroencephalogram (EEG), and fundus photography. Blood samples were collected, and genomic DNA was extracted. Family members, when available, were also recruited for segregation and haplotype analysis. In addition, we have performed a clinical comparison with the previously diagnosed ST‐1 case, which was confirmed through genetic diagnosis.

### Molecular genetics

Whole exome sequencing (WES) was carried out on 18 patients with PMA and 202 individuals with ataxia, utilizing methodologies referenced from prior reports.^14^ The remaining 13 PMA patients underwent Sanger sequencing of the *NEU1* gene. Sanger sequencing was performed on the entire coding regions and the intron‐exon boundaries of the *NEU1* gene (NM_000434.3) by using the ABI 3700 automated sequencer (Applied Biosystems). We used PCR primers as previously described.^1^

### Plasmids and mutagenesis

Human wild‐type Neu1 and c.951_968delGGTAGCTGCAGGAGCTGT (p.318‐323delVAAGAV) Neu1, along with PPCA cDNA, were synthesized with a Myc tag and HA‐tag at the C‐terminus, respectively. These sequences were then inserted into a pCDNA3.1 vector (Tsingke). The mutations c.517T>G (p.W173G) and c.544A>G (p.S182G) were introduced into the expression constructs using a Fast Mutagenesis Kit (Vazyme).

### Cell culture and transfection

HEK293T cells were purchased from the Cell Bank of Chinese Academy of Sciences (www.cellbank.org.cn). HEK293T cells were cultured in DMEM (Gibco) growth medium, containing 10% FBS (Gibco) and 1% penicillin–streptomycin (Gibco). After reaching 80% confluence, HEK293T cells were transfected with 2.5 μg vector or Neu1 wild‐type or mutant plasmid using the Lipofectamine 3000 reagent (Invitrogen, L3000‐008) following the manufacturer's manual. The cells were collected for further analysis 48 h after the transfection. For the NEU1 enzyme, PPCA was cotransfected, which is known to be associated with the NEU1 protein and β‐galactosidase as a complex in the lysosomes to maintain the sialidase activity.^15^

### Western blot

Forty‐eight hours post‐transfection, the cells were lysed and extracted protein. The protein extracts were then resolved using 4%–12% w/v SDS‐PAGE gradient gels and transferred to nitrocellulose (NC) membranes. The NC membranes were blocked with 5% w/v bovine serum albumin before incubation with primary antibodies overnight at 4°C. The following primary and secondary antibodies were used in this experiment: anti‐Myc tag (1:1000, CST, 2276S), anti‐Vinculin (1:10,000, Abcam, ab129002). Horseradish peroxidase (HRP)‐labeled goat anti‐mouse IgG (H + L) (1:3000, Beyotime, A0216), and HRP‐labeled goat anti‐rabbit IgG (H + L) (1:3000, Beyotime, A0208).

### Neuraminidase activity assay

The neuraminidase (NA) activity was measured by a fluorometric assay with substrate 20‐(4‐methylumbelliferyl)‐αd‐N‐acetylneuraminic acid (MUNANA) (ab138888, Abcam). Forty‐eight hours post‐transfection, the cells were lysed with 0.1% Triton X‐100. After centrifugation at 16,000 × *g* at 4°C for 10 min, the supernatants were subjected to an NA assay at 37°C for 1.5 h. Reaction products were measured at 365 nm for excitation and 450 nm for emission.

### Sialic acid assay

The total sialic acid (TSA) and free sialic acid (FSA) were measured by a fluorometric assay with sialic acid assay kit (MAK314, Sigma). Briefly, to determine TSA, urine of healthy controls (HCs) and patients was hydrolyzed to release bound sialic acid. In a microcentrifuge tube, mix 20 μL of urine, 40 μL of ultrapure water, and 40 μL of Hydrolysis Reagent. Heat at 80°C for 60 min, add 25 μL of 10% TCA and centrifuge at 14,000 rpm for 10 min. Supernatant was transferred and labeled as ‘TSA’. To determine FSA, directly precipitate protein by mixing 40 μL of urine and 10 μL of 10% TCA. Vortex and centrifuge at 14,000 rpm for 10 min. Supernatant was transferred and labeled as ‘FSA’. After oxidation and color Reaction, the final reaction mixture is transferred into wells of a black, flat‐bottom 96 well plate. Read fluorescence intensity (λex = 555/λem = 585 nm).

### Haplotype analysis

The haplotype associated with *NEU1* c.544A>G mutation was first estimated using genotype data generated using HumanOmniZhongHua‐8 Bead Chip (Illumina) in 5 consanguineous families (Family A‐E, the red dots mark individuals selected for genotyped). Raw data were processed and quality‐checked using the Genome Studio software (Illumina). All individuals passed the 99% call rate threshold and were included in the following analysis. Linkage‐disequilibrium pruning was performed using PLINK v1.07^16^ and keeping SNPs with minor‐allele frequency (MAF) >0.05, missingness <10%, and *R* ^2^ <0.50 by using a window size of 50 SNPs and a 5 SNP overlap between windows. Finally, the dataset was thinned to include 0.1‐cM‐spaced markers. Fourteen tag SNPs were selected for haplotype reconstruction within a 2.3 Mb region surrounding the mutation (8 sites upstream and 6 sites downstream): rs1736936, rs2072107, rs2844714, rs9394023, rs6923313, rs2248373, rs2295663, rs9368699, rs494620, rs486416, rs1048709, rs6941112, rs2856448, and rs17421624. The 14 SNP genotypes were determined by Sanger sequencing for patients with homozygous c.544A>G mutation in *NEU1*.

### Statistical analyses

Statistical analyses were performed using GraphPad Prism (GraphPad Software). Error bars represent standard deviations (SD) as indicated in the figure legends. Mann–Whitney test was used to compare three or more columns of data. Differences between two groups was performed using a two‐tailed, unpaired Student's *t* test. Data represent means ± SD. *p < 0.05; **p < 0.01; ***p < 0.001; ****p < 0.0001; ns, not significant. *p* < 0.05 was considered statistically significant.

## Results

### Identification of biallelic mutations in *NEU1*

**Figure 1:**
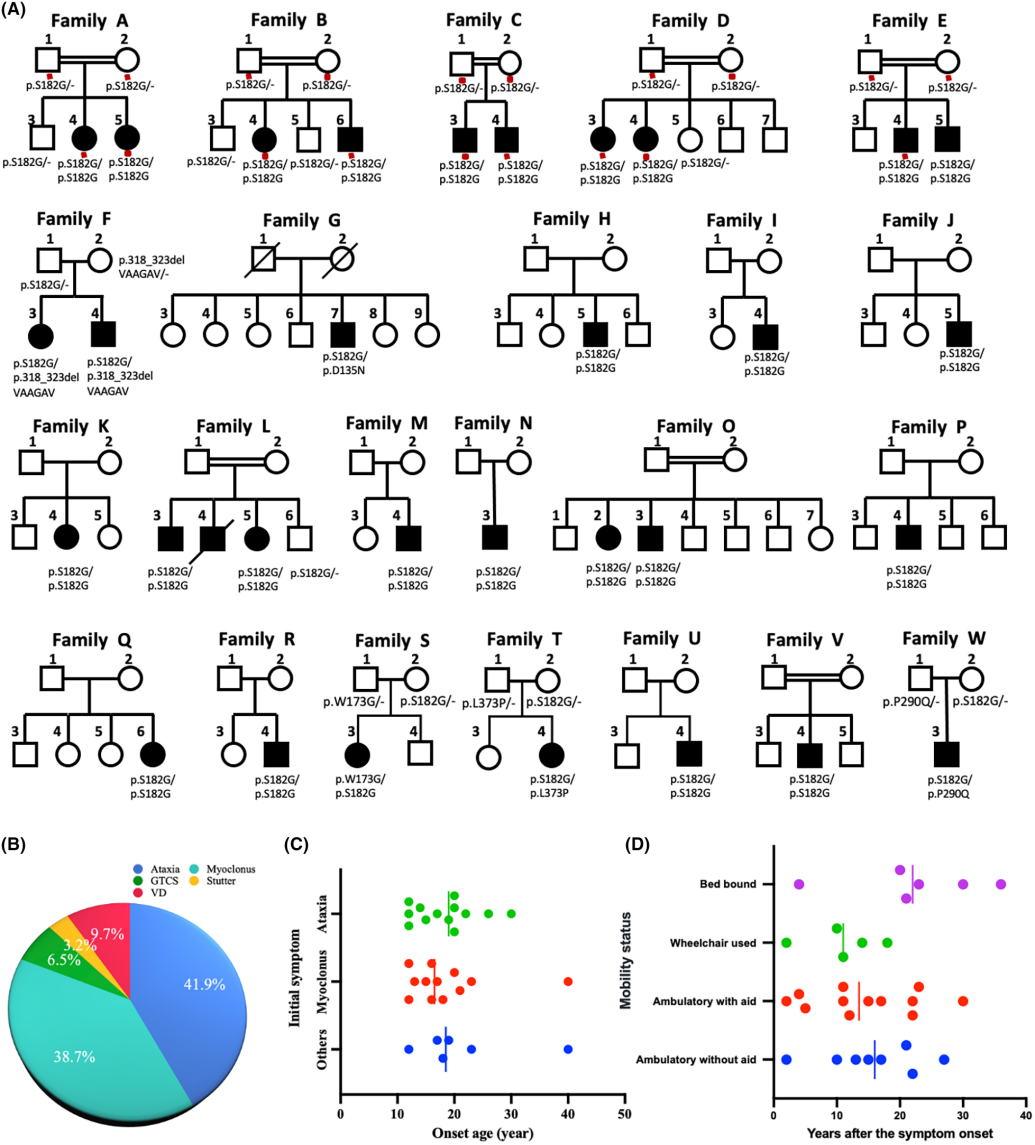
Family pedigree and clinical profiles of patients with type I sialidosis. (A) Pedigrees of 23 ST‐1 families with *NEU1* mutations. Men are represented by squares, women by circles. Filled, empty and slashed symbols indicate affected individuals, unaffected individuals, and deceased individuals, respectively. Double lines indicate parental consanguinity. ‐, reference allele. The red dots indicate genotyped individuals. (B) Distribution of initial symptoms in ST1 patients. (C) Age of onset distribution for patients with different initial symptoms. (D) Relationship between mobility states and disease duration.

We initially recruited 31 patients considered to adhere to the criteria of PMA. Among them, 26 patients were found to carry two pathogenic mutations in the *NEU1* gene. Since five patients in the first cohort initially presented with ataxia, we further included 202 patients presenting with ataxia. Surprisingly, five of these 202 patients (2.5%) were found to have the c.544A>G mutation and genetically diagnosed with ST‐1. Finally, thirty‐one patients from 23 unrelated families with biallelic mutations in *NEU1* were included in this study. Sixteen were familial cases and 15 were sporadic (Fig. 1A). Six different *NEU1* variants were detected in the 31 patients, among which four variants had previously been documented as causative for ST‐1. The other two were novel, including c.951_968delGGTAGCTGCAGGAGCTGT (p.318‐323delVAAGAV), and c.517T>G (p.W173G). 80.6% patients had c.544A>G (p.S182G) mutation homozygous (Fig. 3A). The deletion was located near Asp‐box motifs, which are essential for neuraminidase function and lead to the loss of six amino acids at a highly conserved site. All of these mutations were confirmed to co‐segregate with the disease phenotype. All the patients with biallelic mutations in *NEU1* were genetically diagnosed with ST‐1.

### Clinical features of Chinese patients with ST‐1

The clinical features of patients with ST‐1 are summarized in Table 1. The most common initial symptoms were ataxia (41.9%), affecting 13 patients, and myoclonus (38.7%), affecting 12 patients (38.7%). These were followed by blurred vision (9.7%), seizures (6.5%), and speech disorders (3.2%, Fig. 1B). The onset age was 16.5 years (interquartile range [IQR]13.5–20.8) for myoclonus, 19.0 years (IQR: 13.0–21.0) for ataxia, and 18.5 years (IQR: 15.8–27.3) for other symptoms, with no significant differences among them (Fig. 1C). Myoclonus can be seen in all of the patients and began on average at age 19.0 years (range: 12–55 years). It is mostly triggered by action or even the intention to move and worsened with external stimuli and emotional stressors. During relaxation, myoclonus was minimal and almost completely absent in majority of our patients. However, the severity of myoclonus differed, even among members of the same family (see Fam L‐3, 5, Video S1). And there were five patients (Fam B‐4, Fam C‐4, Fam E‐4, Fam L‐3 and Fam P‐4) experienced periods of severe status myoclonus which was characterized by continuous generalized myoclonus for hours to a day at a time. Myoclonus could be partially controlled by drugs including clonazepam, valproic acid, or levetiracetam.

**Table 1:** Clinical features of patients with type I sialidosis in our study.

Patients	Age	Gender	Symptom onset age	Family history	Initial symptoms	Myoclonus AAO (years)	Myoclonus	Seizures	Ataxia	CRS	OCT	Current state, AAO(y)
A‐4	36	F	12	+	Myoclonus	17	+	+	+	−	NA	Wheelchair used, 30
A‐5	35	F	14	+	Ataxia	15	+	−	+	−	NA	Wheelchair used, 28
B‐4	40	F	17	+	Myoclonus	17	+	+	+	NA	NA	Bed bound, 37
B‐6	36	M	15	+	Ataxia	17	+	+	+	+	LMT and pRNFL thinning	Ambulatory without aid
C‐3	38	M	21	+	Myoclonus	21	+	+	+	−	NA	Ambulatory without aid
C‐4	34	M	17	+	GTCS	17	+	+	+	−	NA	Bed bound, 21
D‐3	40	F	18	+	Myoclonus	18	+	−	+	−	NA	Ambulatory with aid
D‐4	38	F	16	+	Myoclonus	16	+	+	+	−	NA	Ambulatory with aid
E‐4	43	M	13	+	Myoclonus	13	+	+	+	−	NA	Bed bound, 34
E‐5	30	M	19	+	Stutter	20	+	−	+	NA	NA	Ambulatory with aid
F‐3	17	F	12	+	GTCS	12	+	+	+	+	NA	Ambulatory with aid
F‐4	16	M	12	+	Ataxia	12	+	+	+	NA	NA	Ambulatory with aid
G‐7	52	M	30	−	Ataxia	33	+	−	+	−	LMT and pRNFL thinning	Wheelchair used, 40
H‐5	37	F	19	−	Ataxia	19	+	−	+	NA	NA	Wheelchair used, 30
I‐4	30	M	15	−	Myoclonus	15	+	+	+	NA	Normal	Ambulatory without aid
J‐5	30	M	20	−	Ataxia	22	+	−	+	−	NA	Ambulatory without aid
K‐4	33	F	20	−	Ataxia	26	+	−	+	−	Normal	Ambulatory without aid
L‐3	44	M	17	+	Ataxia	17	+	−	+	−	LMT and pRNFL thinning	Bed bound, 40
L‐5	40	F	18	+	VD	24	+	−	+	−	Normal	Ambulatory without aid
M‐4	38	M	23	−	Myoclonus	23	+	+	+	−	NA	Ambulatory with aid
N‐3	50	M	20	−	Ataxia	23	+	−	+	NA	NA	Ambulatory with aid
O‐2	63	F	40	+	VD	55	+	−	+	−	NA	Ambulatory with aid
O‐3	57	M	40	+	Myoclonus	40	+	−	+	−	NA	Ambulatory with aid
P‐4	54	M	16	−	Myoclonus	25	+	+	+	−	NA	Bed bound, 52
Q‐6	54	F	20	−	Myoclonus	29	+	+	+	−	NA	Bed bound, 50
R‐4	50	M	23	−	VD	23	+	−	+	−	NA	Ambulatory without aid
S‐3	14	F	12	−	Ataxia	12	+	−	+	NA	NA	Ambulatory without aid
T‐4	14	F	12	−	Myoclonus	12	+	−	+	NA	NA	Ambulatory with aid
U‐4	48	M	26	−	Ataxia	26	+	−	+	NA	NA	Ambulatory with aid
V‐4	33	M	22	−	Ataxia	22	+	−	+	−	NA	Ambulatory with aid
W‐3	14	M	12	−	Ataxia	12	+	−	+	NA	NA	Wheelchair used, 14

AAE, age at examination; AAO, age at onset; CRS, cherry‐red spots; F, female; GTCS, generalized tonic–clonic seizure; LMT, Localized macular thinning; M, male; NA, not available; OCT, optical coherence tomography; pRNFL, peripapillary retina nerve fiber layer; VD, visual defect; y, years.

Ataxia was also observed in all the patients in early stage of the disease. It could manifest as gait instability and poor performance in precise, coordinated movements, which also happened as a result of myoclonus. Clinical distinction of incoordination due to cerebellar signs versus action myoclonus is difficult. Generalized tonic–clonic seizures (GTCS) were reported in 13/31 (41.9%) patients, and the average age of onset of GTCS was 23.7 years (range: 12–51 years). All of the patients received anti‐epileptic drugs soon after GTCS onset, and GTCS could be well‐controlled by anti‐epileptic drugs, with less than one attack per year.

**Figure 2:**
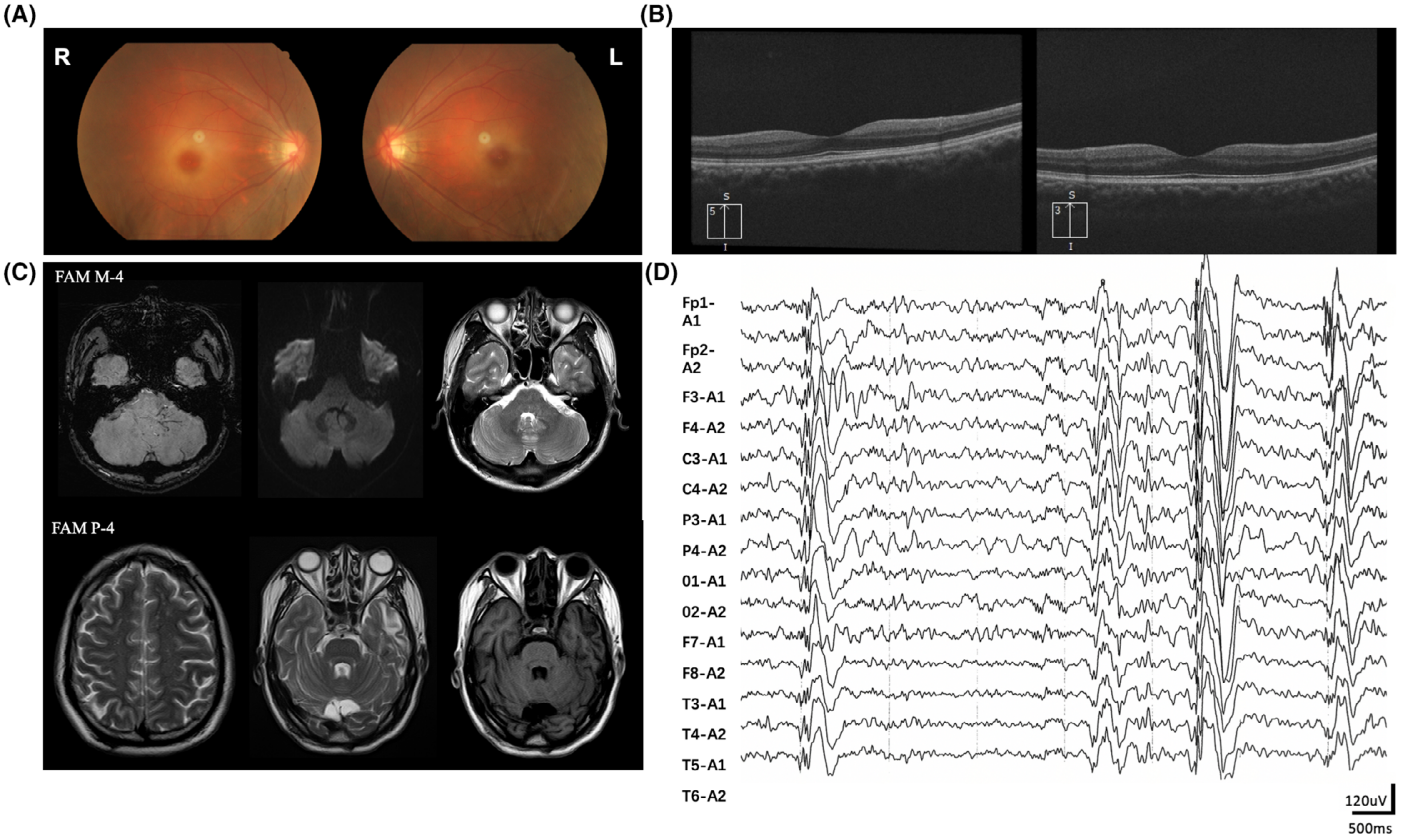
Auxiliary examination results of patients. (A) Color fundus photographs of the patient Fam F‐3 showed bilateral cherry‐red spots in the macular. (B) Optical coherence tomography of patient Fam B‐6 and Fam L‐3 showed thinning of the retinal nerve fiber layer. (C) Brain MRI of the patient Fam M‐4 exhibited several linear dilated veins in the left cerebellar hemisphere and pontic arm. The diffuse weighted imaging (DWI) sequence revealed flow shadows. On T2‐weighted image (T2WI), the lesion was illegible. Brain MRI of Patient Fam P‐4 showed several spot‐like T2WI hyperintensities in the bilateral frontal and parietal lobes, as well as patchy T2WI hyperintensities and T1‐weighted image hypointensities in the left temporal lobe. (D) EEG of the patient Fam P‐4 showed generalized polyspike and polyspike‐slow waves.

At last follow‐up, eight of the patients (median duration: 16.0 years, IQR: 10.8–21.8) were still able to walk cautiously without aid, but none of them were able to maintain regular jobs. Twelve patients (median duration: 13.5 years, IQR: 6.5–22.0) used walking aids in daily life. Five patients (median duration: 11.0 years, IQR: 6.0–16.0) used wheelchair, but they were able to do look after own affairs independently. And six patients (median duration: 22.0 years, IQR: 16.0–31.5) were bedridden and required constant nursing care and attention (Fig. 1D). The patients' mobility did not show a significant correlation with the duration of the disease; however, as the disease progressed, patients gradually lost their ability to move independently. None of the patients showed cognitive impairment except for patient Fam F‐4, who was diagnosed with autism at 5 years old. Macular cherry‐red spots were documented only in 2/21 (9.5%) patients (Fig. 2A), although 18/31 (58.1%) patients complained about visual impairment. Optical coherence tomography was performed on six patients, revealing localized thinning of the retinal thickness in the macular region and thinning of the retinal nerve fiber layer around the optic disc in three patients: Fam B‐6, Fam G‐7, and Fam L‐3 (Fig. 2B). The other three patients exhibited normal retinal findings.

### Brain MRI and electroencephalogram

Brain MRI was available for 14 cases, with disease duration ranging from 2 to 38 years, and the average duration was 18.8 ± 9.0 years. Apart from notable findings in individuals Fam M‐4 and P‐4, the majority of the others were mostly normal (Fig. 2C). Fam M‐4 was presumptively diagnosed with venous hemangiomas, while Fam P‐4 exhibited imaging findings of punctate hyperintensities in the bilateral frontal and parietal lobes and patchy abnormalities in the left temporal lobe. EEG data were available for 17 cases, revealing varying degrees of diffuse paroxysmal activity, including polyspike complexes (Fig. 2D).

### Clinical and genetic comparison with previous studies

Comparing our patient with several previous studies from Mainland China and Taiwan that have clear genetic diagnoses and comprehensive clinical data,^3^, ^5^, ^17^, ^18^ as shown in Table 2. The previous studies included a total of 32 patients, comprising 13 females and 19 males. We found that the age of onset in our patient was later than in the previous studies (18.0 ± 7.1 years vs 14.0 ± 6.6 years). There are various types of initial clinical symptoms, but in our patient, the primary symptoms were myoclonus and ataxia, whereas in the previous reports, myoclonus and GTCS were more common. Most patients exhibited myoclonus and ataxia, but the prevalence of seizures and CRS in our study was lower than in the previous studies (41.9% vs 83.8%, 9.5% vs 46.9%). Additionally, the majority of patients carried the c.544A>G mutation (100% in our study compared to 87.5% in previous studies).

**Table 2:** Comparison of clinical and genetic data across ST‐1 case series.

	Our patients	Published cases
Patients, n, F/M	31 (13/18)	32 (13/19)
Age of onset disease (years)	18.0 ± 7.1	14.0 ± 6.6*
Initial symptoms		
Myoclonus	12 (38.7%)	11 (34.4%)
Ataxia	13 (41.9%)	3 (9.4%)
GTCS	2 (6.5%)	5 (15.6%)
Stutter	1 (3.2%)	0 (0%)
Visual defect	3 (9.7%)	4 (12.5%)
Painful paresthesia	0 (0%)	4 (12.5%)
Symmetric distal Neuropathic pain	0 (0%)	1 (3.1%)
Myoclonus	100% (31, 31 Ava)	93.8% (30, 32 Ava)
Seizures	41.9% (13, 31 Ava)	83.8% (30, 32 Ava)***
Ataxia	100% (31, 31 Ava)	87.5% (28, 32 Ava)
Cherry‐red spot	9.5% (2, 21 Ava)	46.9% (15, 32 Ava)**
*NEU1* mutation, c.544A>G	100% (31, 31 Ava)	87.5% (28, 32 Ava)

Ava, available; F, female; GTCS, generalized tonic–clonic seizure; M, male.

* *p* < 0.05.

** *p* < 0.01.

*** *p* < 0.001.

### Decreased Neu1 mutant protein expression and enzyme activity elevate urinary sialic acid levels

**Figure 3:**
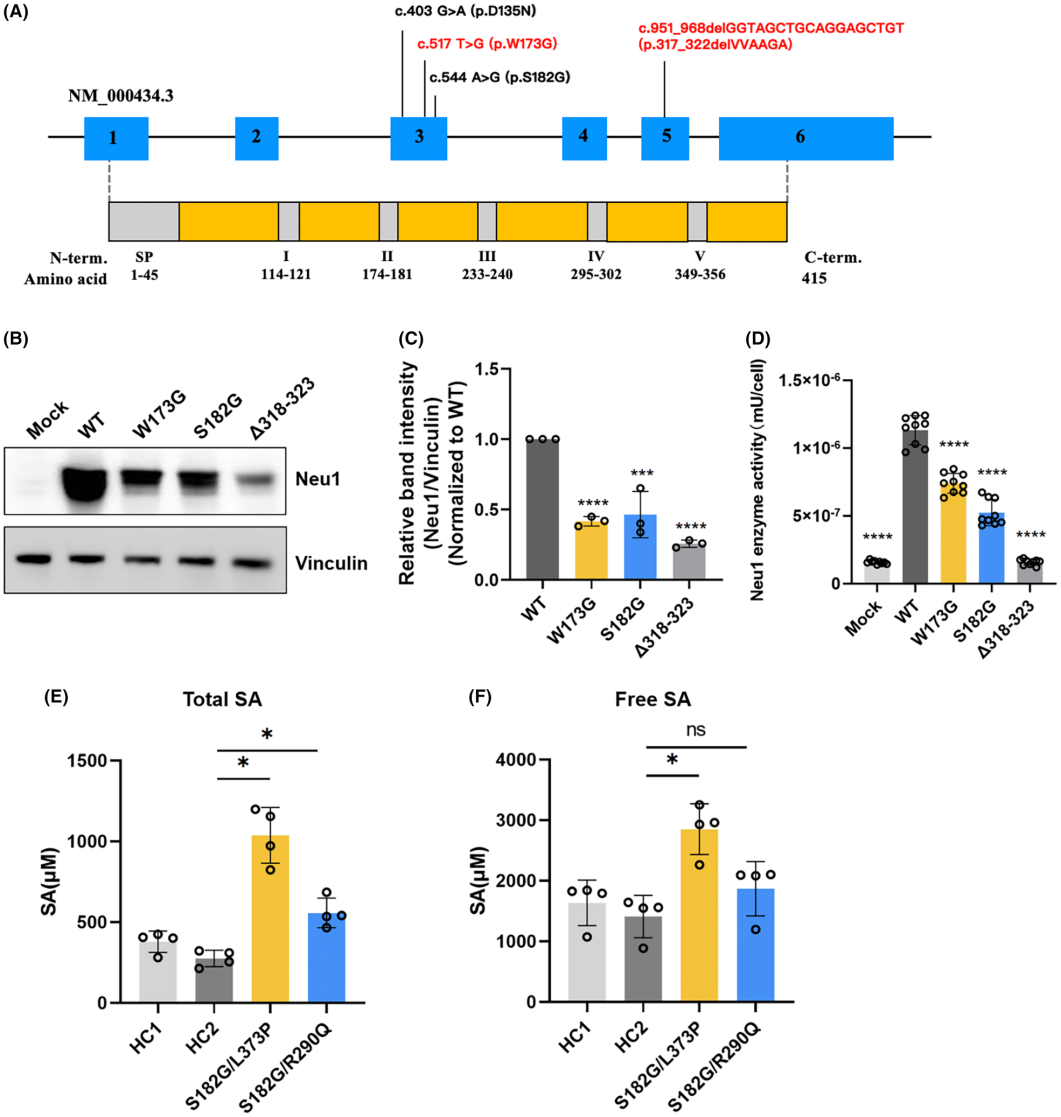
Molecular and biochemical characteristics of sialidosis patients. (A) Distribution of mutations within the *NEU1* gene and primary structure of neuraminidase. Two novel mutation (c.951_968delGGTAGCTGCAGGAGCTGT/p.318‐323delVAAGAV and c.517T>G/p.W173G) (labeled in red) and two previous reported mutations (c.544A>G/p.S182G and c.403G⟩A/p.D135N) (labeled in black) were identified. (B) Western blot analysis showed a significant decrease in protein expression for all Neu1 mutants compared to WT. (C) Quantification of Neu1 protein levels confirmed the reduction in expression for the mutant variants. (D) Neuraminidase activity assay demonstrated significantly reduced enzyme activity in cells expressing Neu1 mutants compared to WT. (E) Elevated total sialic acid levels were observed in the urine of two ST‐1 positive patients compared to healthy controls (HCs). (F) Free sialic acid levels were mildly elevated in patients with compound heterozygous mutations (c.544A>G, p.S182G and c.1118T>C, p.L373P).

To test whether the two novel mutations, c.951_968del and c.517T>G, retained the ability to hydrolyze terminal sialic acid residues and neuraminic acids, a neuraminidase assay was performed. Prior to this, Neu1 protein expression was examined in HEK293T cells transfected with wild‐type Neu1 (WT) and the two novel Neu1 variants, as well as the c.544A>G variant (Fig. 3B). Quantification analysis revealed a significant decrease in protein expression in all mutant Neu1 variants compared to the WT Neu1 protein (Fig. 3C). Subsequently, the neuraminidase assay conducted on HEK293T cells transfected with the Neu1 variants showed significant reductions in enzyme activity for the Neu1 mutants compared to the WT (Fig. 3D).

Absence of sialidase results in abnormal intracellular accumulation and urinary excretion of sialyloligosaccharides, and causes an increase in the level of conjugated sialic acid bound to glycoproteins and glycolipids. Moreover, accumulation of free and bound sialic acid were observed in sialidosis fibroblast strains.^19^ To investigate this, a sialic acid assay was performed on the urine of two ST‐1 positive patients and two healthy controls (HCs). The results showed that total sialic acid levels, measured after acid hydrolysis, were significantly elevated in the patients compared to the HCs (Fig. 3E). Additionally, free sialic acid levels were mildly elevated in patients with compound heterozygous mutations (c.544A>G, p.S182G and c.1118T>C, p.L373P) (Fig. 3F).

### The *NEU1* c.544A>G mutation is a founder variant

**Figure 4:**
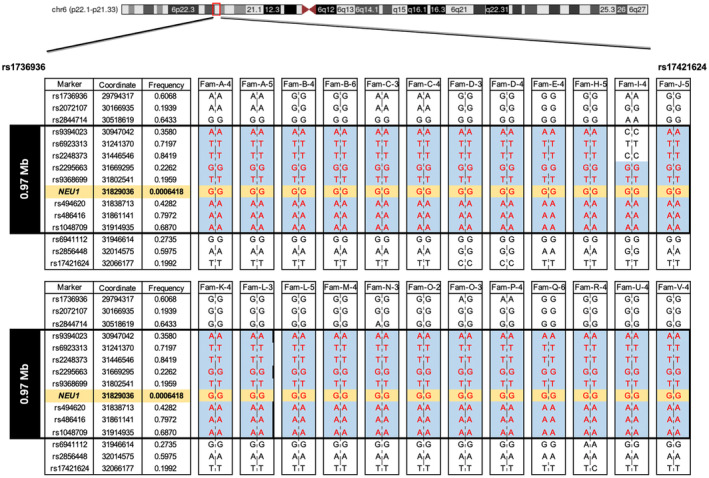
Identification of the founder haplotype. Haplotype analysis of 14 SNP markers flanking the *NEU1* gene in 18 pedigrees harboring the *NEU1* c.544A>G (chr6:31829036) homozygous mutation (in orange) showed the 0.97 Mb core haplotype between rs9394023 and rs1048709 (except for Fam‐I‐4). The linked haplotype is labeled with blue and genotypes that could be fully phased are shown in red. Fourteen SNPs are showed in forward orientation. Allele frequencies of SNPs in East Asian population from gnomAD V2.1.1 (include only Genomes) were shown. Genomic locations are from the Genome Reference Consortium Human Build 37.

All of the patients and their ancestors were born to long‐term resident families of Fujian, a coastal province in southeast China. To investigate the founder effect of the *NEU1* c.544A>G mutation in our case series, we first estimated the haplotype in five consanguineous families (Family A‐E, the red dots mark individuals selected for genotyped). Then we genotyped 14 tag SNPs in patients with homozygous c.544A>G mutation in *NEU1*. We found that these ST‐1 families shared a common haplotype linked to *NEU1* c.544A>G, covering a region of 0.97 Mb between loci rs9394023 and rs1048709 (Fig. 4), suggesting the presence of a founder effect for the c.544A>G mutation. The allelic carrier frequency of this variant in the East Asian control population is 0.001361 based on an allelic count of 27 out of 19832 genomes in the gnomAD v2.1.1; no carrier of this allele was observed in any of the other populations in the database. And a c.544A>G carrier was found among the 200 Indigenous healthy unrelated controls (1/400 alleles).

## Discussion

In this study, we report a Chinese cohort of 31 patients with ST‐1 from 23 unrelated families; making this the largest ST‐1 cohort with detailed phenotypic and genotypic characterization reported to date. Major findings of the present study including the following: (a) the phenotype in our cohort resembles PMA without cherry‐red spots, and isolated ataxia is noted as one of the most common initial symptoms; ST‐1 can be underdiagnosed among both PMA and ataxia patients. (b) All 31 cases have the same mutation (c.544A>G) and haplotype analyses confirm a founder effect; the regional frequency of c.544A>G mutation is strikingly high.

Consistent with previous studies,^1^, ^3^, ^5^, ^6^, ^20^ the most common initial chief complaint and symptoms of patients in our cohort were ataxia and myoclonus. It is worth noting that ataxia could appear much earlier than myoclonus in our study. Previous studies have predominantly identified myoclonus and GTCS as the initial symptoms.^3^, ^5^, ^17^, ^18^ Ataxia and myoclonus were initially noticed around the age of 19.3 and 21.2 years, respectively, among our 13 patients with initial complaints of ataxia. Moreover, worsening of myoclonus could lead to frequent falls, which were often documented as gait instability. All the above points led to a high risk of missed diagnosis in patients with ataxia. In our study, five patients (2.5%) were positive for the *NEU1* gene screen in a cohort of 202 undiagnosed ataxia patients. Thus, when a patient with ataxia tests negatively in screening for the relatively common spinocerebellar ataxias types, *NEU1* genetic screening should be considered.

As the disease progresses, patients experience varying degrees of mobility loss. In our study, we found no significant correlation between the duration of the disease and the patients' mobility. Notably, even among patients with the same mutation site, the time to loss of mobility varied significantly. For example, patients with the c.544A>G homozygous mutation displayed different disease progressions: Patient C‐4 became bedridden at age 21 after a 4‐year disease course, while Patient E‐3 became bedridden at age 34 after a 21‐year disease course. This inconsistency suggests that factors other than the duration of the disease and the mutation site may influence the progression of mobility loss. To better understand these variations, larger sample sizes and further investigation into potential protective factors are needed.

ST‐1 has been referred to as “cherry‐red spot myoclonus syndrome,”^4^ and previous studies emphasized that cherry‐red spots should be sought when sialidosis is suspected clinically.^21^ However, only two patient (2/21, 9.5%) had cherry‐red spots in our cohort. Previous studies involving patients from mainland China and Taiwan have also shown that only 46.9% (15/32) of patients exhibit CRS. The essence of macular cherry‐red spots is that accumulation of storage material in the retinal cellular layers makes the fovea relatively transparent and contrasting with surrounding area.^22^ One study suggested that population and ganglion cell loss could explain the absence of a cherry‐red spot.^8^ Thus, we suggest although macular cherry‐red spot is a significant clinical hallmark for ST‐1, its absence cannot rule out the disease.

Sialidosis has been classified under the progressive myoclonic epilepsies (PMEs) spectrum for decades,^23^ which is a group of rare genetic disorders characterized by myoclonus, seizures, and progressive neurological deterioration, particularly dementia and ataxia.^21^ However, seizure was only observed in 41.9% of our patients, and all patients presented with normal cognition except patient Fam F‐4 combined with autism. Considering the main characteristics of our cohort, we suggest ST‐1 can be classified as PMA. Testing of *NEU1* should therefore be offered to patients with PMA in the absence of other typical clinical futures.

In our cohort of patients who underwent Brain MRI, only two individuals presented with nonspecific imaging findings, both of whom had the homozygous mutation c.544A>G. The vast majority of patients exhibited no notable abnormalities. Prior research on five adolescent patients with ST‐1 revealed that three of them had essentially normal MRI scans of the brain, with their mutations consisting of compound heterozygous variants: c.803A>G/c.239C>T, c.838G>A/c.403G>A, and c.544A>G/c.239C>T, respectively. Conversely, two other adolescent patients, each carrying the genotypes c.544A>G/c.239C>T and c.544A>G/c.1118 T>C, respectively, demonstrated deepened cerebellar sulci, slight atrophy of the cerebellar vermis, and an expanded aqueduct of the midbrain.^3^ Notably, even among patients sharing the same mutation sites, there was a divergence in cranial MRI presentations, suggesting a significant degree of variability in the neuroimaging manifestations of ST‐1, potentially influenced by additional factors. Nevertheless, the predominantly normal brain imaging observed in most patients suggests that the utility of Brain MRI as a diagnostic tool for ST‐1 is somewhat limited.

Pathogenic mutations in the *NEU1* gene are observed across all exons, with mutations in exons 4 and 5 being more prevalent.^3^ In our study, we identified two novel mutation sites: c.517T>G in exon 3 and c.951_968del in exon 5. Loss of function in the NEU1 gene results in decreased sialidase activity. By constructing mutant cell models, we demonstrated that these new mutations lead to reduced levels of Neu1 protein and diminished neuraminidase activity. The mutational spectrum of ST‐1 in China is very different from the reported spectrum for Caucasian populations. No patient with c.544A>G mutation has been identified in Caucasian populations so far. In our cohort, all patients have c.544A>G mutation, and 88.5% patients were homozygous. One study from Taiwan showed similar to our findings.^5^ Given the geographical proximity between Fujian and Taiwan, and the common haplotype identified in our cohort, it is plausible that individuals with *NEU1* c.544A>G in both regions may have arisen from an ancestral founder effect. We also checked the gnomAD database and the ExAC database to find the allele frequency of *NEU1* c.544A>G in East Asians was 0.134% and 0.129%, respectively; this mutation was absent from other populations in both databases. These studies suggest that the allele frequency of *NEU1* c.544A>G in Han Chinese could be unexpectedly high. More Han Chinese and East Asian populations are warranted to test whether the present hypothesis could be generalized to other regions.

In conclusion, our study of the largest ST‐1 Chinese cohort to date reveals a unique clinical and genetic landscape, with isolated ataxia as a common initial symptom and a high prevalence of the c.544A>G mutation suggesting a founder effect in Southeast China. The absence of cherry‐red spots in most patients challenges traditional diagnostic criteria. These insights necessitate a reevaluation of ST‐1 diagnostic protocols. Our findings suggest that ST‐1 should be suspected in Chinese patients with PMA, even in the absence of other typical features, and the common founder variant *NEU1* c.544A>G could be screened first.

## Funding Information

This study has been supported by grants from the National Key Research and Development Program of China grants 2022YFC2703900 (W.J.C.) and 2022YFC2703904 (W.J.C.); from the National Natural Science Foundation of China, including 82025012 (W.J.C.), and U1905210 (W.J.C.); from the Joint Funds for the Innovation of Science and Technology of Fujian Province 2021Y9011 (W.J.C.); from the Fujian Provincial Health Technology projects grants 2022ZD01002 (W.J.C.); from the Major Scientific Research Program for Middle‐aged and Young Scientists of Fujian Province 2021ZQNZD003 (W.J.C.); from the Local Science and Technology Development Project guided by the central government grants 2020L3010 (W.J.C.); from Fujian Medical University Affiliated First Hospital Talent Recruitment Research Initiation grants YJRC4185 (K.Y.); Fujian Natural Science Foundation 2021J01231 (N.Q.C.).

## Conflict of Interest

On behalf of all authors, the corresponding author confirms no conflict of interest.

## Author Contributions

Jingjing Lin, Wan‐Jin Chen, and Kang Yang conceived the idea, designed studies, and supervised the project; Yun‐Lu Li, Hui‐Zhen Su, Yi‐Heng Zeng, Rui‐Huang Zeng, Yu‐Duo Zhang, Ru‐Kai Chen, Nai‐Qing Cai, and Yi‐Kun Chen provided the clinical data; Jingjing Lin, Ru‐Ying Yuan, Jun‐Yi Jiang, Kang Yang, and Xiang‐Ping Yao acquired the literature data and analyzed data; Yun‐Lu Li, Jingjing Lin, Bo‐Li Chen, drafted the manuscript; Ning Wang, Wan‐Jin Chen, and Kang Yang critically revised the manuscript for important intellectual content. All authors read and approved the final manuscript.

## Supporting information


Video S1.

**Video Caption S1**.

## Data Availability

The data that support the findings of this study are available from the corresponding author upon reasonable request.
