# Supplementary material for: Progressive myoclonic ataxia as an initial symptom of typical type I sialidosis with NEU1 mutation
Source: Ann Clin Transl Neurol. 2024 Oct 31;11(11):2998–3009. doi: 10.1002/acn3.52212 (PMC11572746; doi:10.1002/acn3.52212)
Supplement: Supplementary file 2 — Video Caption S1. [file ACN3-11-2998-s001.docx]

Video S1. Segment 1. Patient Fam L-3: examination myoclonus at rest focusing on the face. Segment 2. Patient Fam L-3: myoclonus jerks are seen with arms and hands outstretched. Segment 3. Patient Fam L-3: finger-to-nose tests is impossible because of the action myoclonus and prominent ataxia. Segment 4. Patient Fam L-5: small amplitude myoclonus jerks are seen focusing on hands. Segment 5. Patient Fam L-5: finger-to-nose tests show both action myoclonus and ataxia. Segment 6. Patient Fam L-5 used a soupspoon to bring water from a cup to her mouth: spoon reached her mouth but spilled some water.
